# Oxytocin is associated with PTSD's anxious arousal symptoms in Chinese male earthquake survivors

**DOI:** 10.3402/ejpt.v5.26530

**Published:** 2014-12-09

**Authors:** Chengqi Cao, Li Wang, Richu Wang, Yulan Qing, Jianxin Zhang

**Affiliations:** 1 Key Laboratory of Mental Health, Institute of Psychology, Chinese Academy of Sciences, Beijing, People's Republic of China; 2 Institute of Psychology, University of Chinese Academy of Sciences, Beijing, People's Republic of China

**Keywords:** PTSD, oxytocin, phenotypic model, association study

## Abstract

**Background:**

Posttraumatic stress disorder (PTSD) is a complex and severe mental disorder triggered by exposure to an extraordinarily traumatic event. Human and animal studies have implied the functional role of the oxytocin system in the development of PTSD (Cochran, Fallon, Hill, & Frazier, 2013; Koch et al., 2014; Olff, 2012). Specification of the role of the oxytocin system in the emergence and progression of PTSD symptomatology would provide evidence to inform both theory and clinical practice.

**Methods:**

This study examined the association between oxytocin serum levels and PTSD symptoms. A total of 106 Chinese male adults who suffered from the deadly 2008 Wenchuan earthquake participated in this study. PTSD symptoms were measured with PTSD Checklist for DSM-5 (PCL-5), and serum oxytocin level was determined with ELISA oxytocin kits.

**Results:**

The mean score on the PCL-5 was 19.30 (SD=14.50, range: 1–65) in this sample. The mean oxytocin level was 101.59 pg/ml (SD=55.89, range: 31.50–286.71). The results indicated that although the oxytocin was not associated with total PTSD symptoms, it was associated with PTSD's anxious arousal symptoms.

**Conclusion:**

These findings support that the oxytocin may play an important functional role in the development of PTSD and contribute to the extant knowledge on the genetic basis of the PTSD symptoms.
